# Effects of Infantile Repeated Hyperglycemia on Neuronal Density of Hippocampus and Pentylentetrazol Induced Convulsions in Male Wistar Rats

**Published:** 2012

**Authors:** Malihe Moghadami, Ali Moghimi, Razieh Jalal, Morteza Behnam-Rasouli, Naser Mahdavi-Shahri

**Affiliations:** 1 *Department of Biology, Faculty of Sciences , Ferdowsi University of Mashhad, Iran*; 2 *Applied Research and Production Centre of Lab Animals, (Radman Pajoohan Jam Co.,), Roshd Centre of Ferdowsi University of Mashhad(FUM), Mashhad, Iran*; 3 *Applied Research Centre for Neurofeedback and Neurobehavioral Sciences"(Aren) , Deptartment of Biology, Faculty of Science, Ferdowsi University of Mashhad (FUM), Mashhad, Iran*; 4 *Department of Chemistry, Faculty of Sciences , Ferdowsi University of Mashhad, Mashhad, Iran*

**Keywords:** Convulsion, Hippocampus, Hyperglycemia, Neuronal density, PTZ, Stereological method

## Abstract

**Objective(s):**High blood glucose induces molecular, cellular, morphological and behavioral changes in the brain. Metabolic disturbances, contribute to the hippocampus injury and development of partial focal seizures. The aim of this study was to investigate the effects of infantile repeated hyperglycemia on neuronal density of hippocampal CA_3_ region in newborn Wistar male rats and its effect on chemoconvulsant pentylentetrazol (PTZ) induced generalized seizures in adults.

**Materials and Methods:**Ten days old male Wistar rats were randomly divided into 2 groups (n=20 for each): hyperglycemic and control. Hyperglycemia was induced by intraperitoneal injections of 2 g/kg dextrose solution, twice a day, for 2 weeks. Control animals received saline solution in the same manner. Blood glucose was regularly measured. After that, the brains of rats from each group (n=10) were removed for histological analysis of the CA_3_ region of hippocampus by stereological method. Other animals (n=10) were kept to grow older. Afterwards, seizure was induced in hyperglycemic and control adult rats, by an intraperitoneal injection of 45 mg/kg PTZ solution and then latency of convulsions onset and severity of seizures for each group were recorded.

**Results:**Results showed that hippocampal neuronal density decreased significantly and susceptibility to PTZ induced convulsions increased in experimental animals.

**Conclusion:**The result determined that repeated increments in daily blood sugar levels in infantile period may damage neuronal structures of hippocampus and also make adults more susceptible to PTZ induced convulsions in adulthood period.

## Introduction

Hyperglycemia is one of the most common and most serious metabolic disorders (1, 2). Several studies have demonstrated the effects of hyperglycemia on neurophysiologic alterations (3), cognitive abnormalities (4) and functional impairments in the CNS (5-9). Previous investigations showed that abnormal glucose levels, whether too high or too low, can cause seizures. Clinical studies show that adults with hyperglycemia have an increased predisposition to seizure (10-14). Also, several data indicated that, in the adult rats, high glucose concentrations are associated with proconvulsant effects (15). In animal experiments, seizure susceptibility has been shown to be related to higher blood glucose levels (15, 16). Such, diabetic hyperglycemia (DH) aggravates seizures and status epilepticus-induced hippocampal damage (17). There are some case reports of people experienced very severe hyperglycemia and an epileptiform convulsion (10, 11, 18). 

Thus, it can be suggested that, focal epileptic seizures can be the first manifestation of a diabetic disorder (19). Also clinically, hyperglycemia-related seizures generally are present as partial motor seizures that may or may not change into generalized form (12, 13, 20). However, we have learned much about anatomical and physiopathological effects related to hyperglycemia and diabetes in adults, while there is little information about effects of hyperglycemia on CNS of newborns. In present research, we studied effects of infantile repeated hyperglycemia on histological characteristics of hippocampal CA3 region in 25-day old rats. Then in the second part of our study, we evaluated the susceptibility of adult rats that survived from infantile repeated hyperglycemia to convulsions using PTZ. Although in previous studies, an association between hyperglycemia and seizures has been suggested, most reports are limited in case reports and focused on very high levels of glucose (11-14). In addition, these case reports did not convey a role of hyperglycemia in seizures. Therefore, at this study, we tried to determine if infantile repeated hyperglycemia aggravates seizure appearance in adult rats that were exposed to high blood glucose in newborn period. 

## Materials and Methods

***Animals***

Forty newborn (10-days old) male Wistar rats, bred in the animal house of Ferdowsi University of Mashhad, Iran, were randomly divided into 2 groups (n= 20 for each): hyperglycemic group and control group. The animals had free access to drinking water and standard rodent diet (Javaneh Khorasan Co., Mashhad). Rats were maintained in standard Plexiglas cages under a constant 12/12 light/ dark cycle and at a temperature of 21-23 °C and 60% of humidity. 

***Hyperglycemia induction and blood glucose measurements***

Hyperglycemia was induced by intraperitoneal injections of dextrose solution provided by Pasteur Institut, Iran ( 50% , 2 g/kg BW, 2 times/ day ) for a period of 15 days in hyperglycemic group (n= 20). Control animals were injected with saline only (n= 20). Blood glucose was measured in blood samples, obtained by tail prick, using a strip operated glucometer (BIONIME), before injection and 30, 60 and 120 min after injection.

***Histological analysis***

At the end of injection period, 25-days old male animals were euthanized by ether inhalation and their brains were removed and prepared for histological analysis. Twenty sections from each animal brain were selected using a uniform random sampling scheme and stained with toluidine blue (Merck). Then numerical density of neurons in CA_3_ region of hippocampus was estimated by stereological methods. Stereology is the study of three-dimensional objects through the interpretation of two dimensional images. This is useful not only because it allows us to study the structure of entire cells and tissues based on thin sections or photomicrographs of sections, but also because it allows us to study this structure quantitatively. Methods of unbiased estimation of the above stereological parameters were described previously in details (21, 22).

***Induction of generalized seizures***

Generalized convulsions were induced by a single intraperitoneal (i.p.) injection of PTZ at a dose of 45 mg/kg BW dissolved in saline in hyperglycemic and control rats at 60 days of age (200-250 g). After that, severity of PTZ induced generalized convulsions (from third stage to the end of seizures) and latency period of generalized tonic-clonic convulsions onset was recorded during 60 min for each group based on our previous work (23).

***Statistical analysis***

All statistical procedures were performed by two-way analysis of variance (ANOVA) with a *post-hoc* Tukey test or Student's t test. A *P*< 0.05 was considered significant. All data were expressed as mean ± SEM. 

## Results

**Figure 1:**
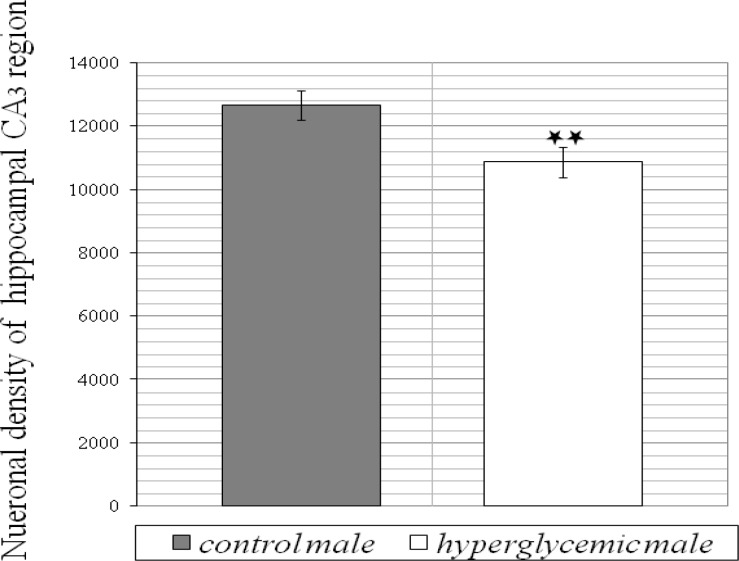
Effects of infantile repeated hyperglycemia on neuronal density of hippocampal CA_3_ region in 25 day old male Wistar rats. Results are expressed as mean±SEM. n=10 for each group. **P< 0.01 compared to control group by Student's t test

**Figure 2:**
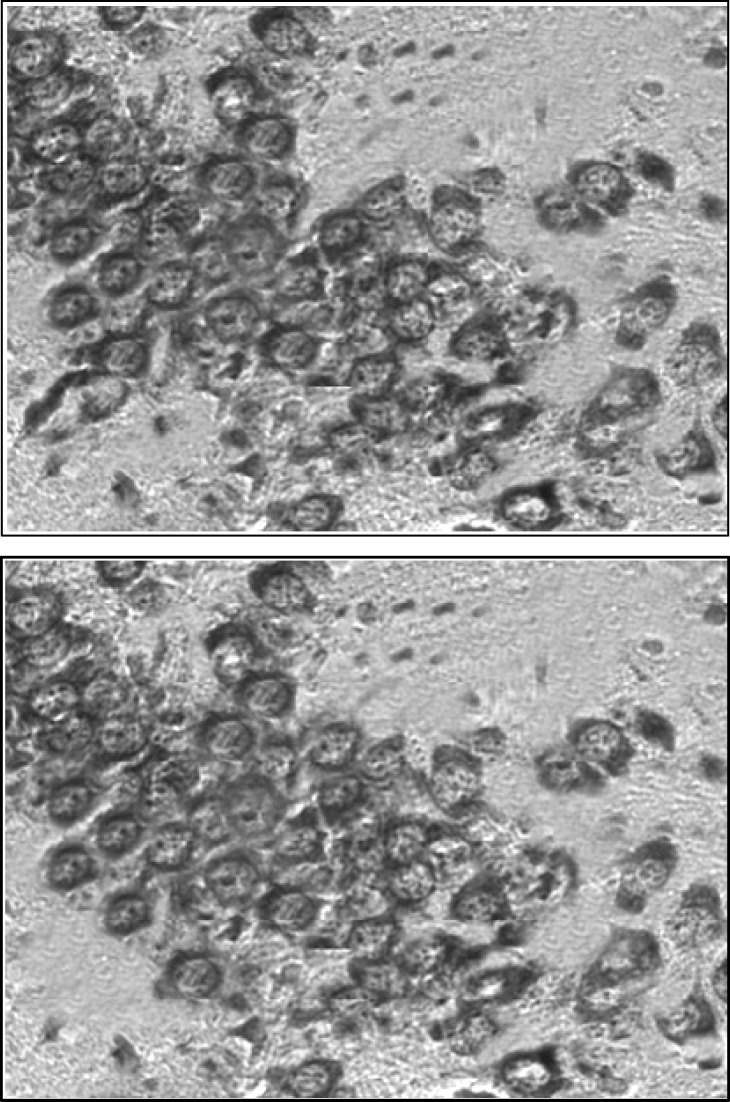
Numerical density of CA_3 _area neurons was estimated by stereological methods as were described in the text. Neuronal density of hippocampal CA3 region in 25 day old hyperglycemic males are decreased in (up image) compared to controls (down image)

**Figure 3:**
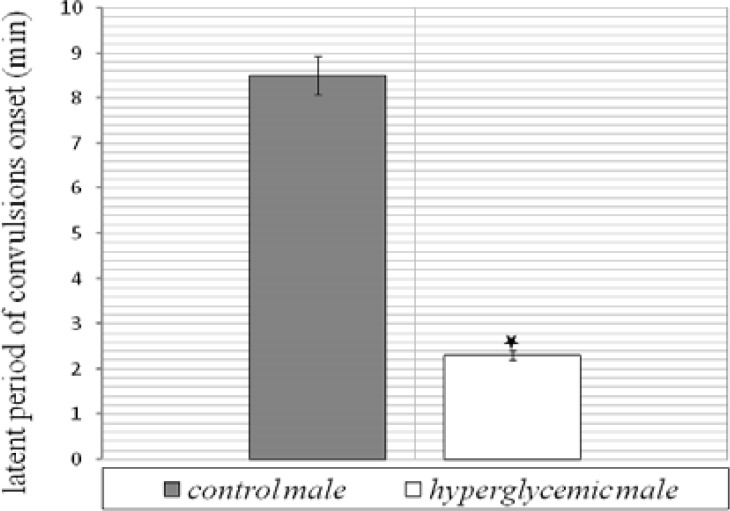
Effect of repeated infantile hyperglycemia on latent period of PTZ induced generalized seizures onset in hyperglycemic and control male groups. Results are expressed as mean ± SEM n=10 for each group. *P<0.05 compared to control group by Student's t test

**Figure 4:**
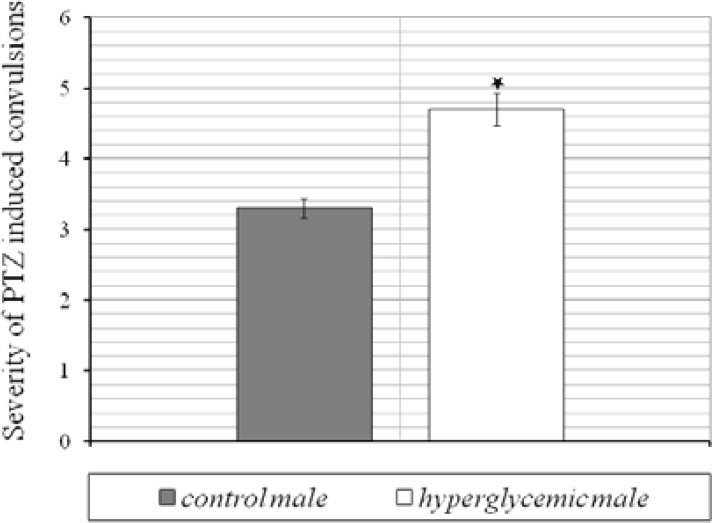
Effect of repeated infantile hyperglycemia on severity of PTZ induced generalized convulsions in hyperglycemic and control male groups. Results are expressed as mean±SEM (n=10, * *P*< 0.05) compared to control group by Student's t test

The levels of blood glucose before and after dextrose solution injection are illustrated in Table 1. As it is shown, 30 min after injection, blood glucose level significantly increased in experimental group and then gradually decreased to normal level, 60 and 120 min after injection. Histological analysis (Figure 1) of brains belong to 25-old days male rats revealed that in comparison to control group, the numerical density of CA_3_ region in hyperglycemic animals significantly decreased *(P*< 0.01). Numerical density was estimated by stereological methods stained with toluidine blue (Figure 2). Images were obtained with light microscope. In addition, hyperglycemia increased the occurrence of seizures induced by intraperitoneal administration of chemoconvulsant PTZ in adult rats. Latent period of generalized seizures occurrence in experimental group reduced compared with control group (*P*< 0.05, Figure 3) and severity of PTZ induced generalized convulsions increased significantly (*P*< 0.05, Figure 4). These results demonstrate that infantile hyperglycemia has a proconvulsant effect on PTZ-induced seizures in the rat. 

**Table 1:** Effect of repeated administration of dextrose solution on blood glucose concentration (mg/dl)

**Day**	**Time 0**	**Time 30**	**Time 60**
**1**	**109.65**	**169.89**	**142.67**
**3**	**126.88**	**187.65**	**146.89**
**5**	**132.76**	**176.54**	**153.76**
**7**	**139.78**	**190.32**	**157.89**
**9**	**145.76**	**224.87**	**158.9**
**11**	**136.65**	**226.87**	**156.43**
**13**	**141.75**	**231.65**	**158.33**
**15**	**146.87**	**234.65**	**159.75**
**Mean**	**135.01**	205.32*	**154.32**
**SEM**	**4.31**	**9.47**	**2.21**

Values are mean ± SEM (n=10, *P*< 0.05)

## Discussion

The present study, demonstrated that repeated increments of blood glucose levels in infantile period can play a remarkable role in appearance of generalized epileptic seizures in adulthood. Moreover, the structure of hippocampus is severely damaged in under-age rats that; a fact that may cause tonic clonic convulsions in adults. Likewise pathophysiology is still under investigation and is probably multi-factorial. 

Previous studies claimed that the temporal lobe is susceptible to seizure activity so, temporal lobe structures, including the hippocampus, have the lowest seizure thresholds in the brain (24). Hyperglycemia-triggered seizures are thought to be due to the increased metabolism of γ-amino butyric acid (GABA) (25). As, increased GABA metabolism lowers the epileptogenic threshold and predisposes it to seizures (20). Homeostasis of electrolytes in the hippocampal extra cellular fluid may be important in preventing seizure activity in aged rats (26). Moreover, over expression of specific glutamate receptors would induce seizures and spontaneous nonsynaptic bursting *in vitro*, and so are associated with cellular changes seen in temporal lobe epilepsy (27). Both *in vivo* and *in vitro* experimental studies suggest that a threshold glucose concentration is necessary to support synaptic transmission. Conversely, it appears that elevated extracellular glucose is associated with neuronal hyper excitability. The importance of glucose balance has been identified in studies demonstrating that hyperglycemia exacerbates ischemia-induced brain damage (16). However, mechanisms underlying the pathogenesis of seizures caused by metabolic disturbances are poorly understood. 

Other hypotheses suggest changes in the hydroelectric balance (28) and alterations in neurotransmitters (29). In addition, epileptiform EEG activity associated with ischemia can contribute to early damage of hippocampal neurons (30). Similarly, some studies revealed that hyperglycemia caused dramatic increase of neuronal alterations and glial cell damage (31), oxidative damage in rat brain (32, 33), and furthermore, enhance oxygen free radical formation (34-36) that will cause damage to nervous tissue (37, 38) and leading to neuronal death (apoptosis), DNA impairment and lipoperoxidation of cell membrane (39). 

Also, hyperglycemia itself is proconvulsant, in both diabetic and normal rats (16). As a result, high blood sugar leads to hyper-excitability of CNS neurons. 

Neurons need a normal level of glucose, their main source of energy, to function correctly. With the brain's overexcited and imbalanced, hyperglycemic seizures can be triggered. In other words, too much sugar makes the neurons work too much, predisposing them to "short circuit," causing a seizure.

Schwechter *et al* (2003) highlighted the link between metabolism and neuronal excitability and emphasized the need for further research on the long-term effects of hyperglycemia on various aspects of brain functions (15). 

As explained, hyperglycemia can affect the neuronal population of hippocampal CA_3_ region, so, there are many suggestions indicating the role of this region in the initiation of seizure. The present study emphasized these opinions. On the one hand hyperglycemia has oxidative effects resulting in cellular death and on the other hand, hippocampal neuronal population decrement will make the brain of rats more susceptible to convulsive effects of PTZ. Thus, susceptibility to clonic and tonic–clonic induced seizures positively correlated with blood glucose concentrations (16), as the increased blood glucose concentration was associated with proconvulsant effects. So, decreased neuronal population induced by infantile repeated hyperglycemia in our studies is in agreement with previous reports (31, 40, 41). Perhaps these results will confirm the cellular structural sensitivity and damage, because of changing in the cellular membranes rigidity as have been suggested (42). 

## Conclusions

The results of our study indicate that repeated hyperglycemia induced obvious cell death in hippocampal CA_3_ region that maybe due to serious changes in metabolic processes, such as abnormal amounts of free radicals formation. And so, hyperglycemia is proconvulsant and might aggravate epileptic seizures in adult rats.
